# How Ecosystem Services Knowledge and Values Influence Farmers' Decision-Making

**DOI:** 10.1371/journal.pone.0107572

**Published:** 2014-09-30

**Authors:** Pénélope Lamarque, Patrick Meyfroidt, Baptiste Nettier, Sandra Lavorel

**Affiliations:** 1 Laboratoire d'Ecologie Alpine, Unité Mixte de recherche 5553, Centre National de la Recherche Scientifique, Université Joseph Fourier, Grenoble, France; 2 Fonds de la recherche scientifique (F.R.S.-FNRS), Louvain-La-Neuve, Belgium; 3 Earth and Life Institute, Georges Lemaître Centre for Earth and Climate Research, University of Louvain, Louvain-la-Neuve, Belgium; 4 Irstea centre de Grenoble, unité de recherche Développement des territoires montagnards, Grenoble, France; USDA-ARS, United States of America

## Abstract

The ecosystem services (ES) concept has emerged and spread widely recently, to enhance the importance of preserving ecosystems through global change in order to maintain their benefits for human well-being. Numerous studies consider various dimensions of the interactions between ecosystems and land use via ES, but integrated research addressing the complete feedback loop between biodiversity, ES and land use has remained mostly theoretical. Few studies consider feedbacks from ecosystems to land use systems through ES, exploring how ES are taken into account in land management decisions. To fill this gap, we carried out a role-playing game to explore how ES cognition mediates feedbacks from environmental change on farmers' behaviors in a mountain grassland system. On a close to real landscape game board, farmers were faced with changes in ES under climatic and socio-economic scenarios and prompted to plan for the future and to take land management decisions as they deemed necessary. The outcomes of role-playing game were complemented with additional agronomic and ecological data from interviews and fieldwork. The effects of changes in ES on decision were mainly direct, i.e. not affecting knowledge and values, when they constituted situations with which farmers were accustomed. For example, a reduction of forage quantity following droughts led farmers to shift from mowing to grazing. Sometimes, ES cognitions were affected by ES changes or by external factors, leading to an indirect feedback. This happened when fertilization was stopped after farmers learned that it was inefficient in a drought context. Farmers' behaviors did not always reflect their attitudes towards ES because other factors including topographic constraints, social value of farming or farmer individual and household characteristics also influenced land-management decisions. Those results demonstrated the interest to take into account the complete feedback loop between ES and land management decisions to favor more sustainable ES management.

## Introduction

Assessing the consequences of ecosystem change on ecosystem services (ES), defined as the outputs of ecosystems [1] from which people derive benefits, is of primary importance. In agro-ecosystems, flows of ES are directly affected by farmers' behaviors and land management decisions [2]. ES stress the need to integrate ecological and social science to study coupled human and natural systems [3], and therefore require to explicitly address the complex feedback loops formed by reciprocal interactions between people and nature [4]. These feedbacks depend on how changes in management affect ES and how, in turn, these changes in ES are perceived by land managers [5]. Nevertheless, while numerous studies consider various dimensions of the interactions between ecosystems and land use via ES, integrated research addressing the complete feedback loop between biodiversity, ES and land use has remained mostly theoretical. Most published frameworks (e.g., [6], [7]) investigate the interactions between ecosystems, ES and human well-being by considering values generated for people, and close the loop by exploring changes and future trends in ES according to scenarios, with possible institutional responses. The full cascade of ES from ecosystem processes to benefits [1] is sometimes considered (e.g., [6], [8]) but the feedbacks effects from ES to human actions and the consequences on ecosystem processes are rarely taken into account [5]. The main research themes in which ES are related to decision-making concern: (i) studies on payments for ES, i.e. financial incentives to sustain management of resources which maintain or enhance ES delivery [9], [10]; (ii) economic valuation is used to raise decision-makers' awareness of the importance of ES through the costs associated with their loss [11], [12]; and (iii) ES mapping as a decision tool for landscape planning [13]. Other studies explored how ES could fit into formal institutional arrangements [14]. However, how people perceive ecosystems and their ability to provide values affects choices about how to manage the environment [6]. Psychology, decision sciences and behavioral economics show that individuals are not necessarily utility maximizers or financially rational [15], and individual preferences are evolving [16]. Economic valuation methods do not adequately address these complexities linked to attitudes and motivational systems, and their effects on behaviors (Kumar and Kumar, 2008). Recent reviews [5], [17] underline the interest of using mental models to explore mechanisms by which individual decisions are made and thereby enhance sustainable management of land and natural resources. A wide range of theories and models based on psycho-social constructs such as attitudes, beliefs and values can help to understand how environmental change can influence decision-making [5], [18]. Two of the most popular theories are the Theory of Planned Behavior (TPB) model [19] and the Value-Belief-Norm Theory (VBN) [20]. Both are based on the premise that individuals' behavior towards the environment is influenced by what they feel and think with respect to the environment. The TPB is based on self-interest and rational choice deliberation, while the VBN focuses primarily on the role of values and moral norms. The main limitation of these theories is that they do not explain the formation of the cognitions (beliefs, values, preferences, attitudes) that are used in a complete feedback loop of decision-making process, and which are crucial to understand adaptation to non-linear and rapid environmental change [5], [18]. Studies that have explored stakeholders' perceptions [21]–[24] or preferences and values [25], [26] in terms of ES have shown the diversity of stakeholders' knowledge and/or values attributed to different ES. Other studies have investigated farmers' decision-making process [27], [28], sometimes taking into account interactions between environmental perceptions and actions [29]–[31], but few of these use the ES framework [32].

To fill this gap, this paper studies how ES are taken into account in land-use decisions in the context of mountain grasslands management. The study area in the Central French Alps is typical of extensive grassland management systems found in drier European mountains, and is mainly composed of permanent grasslands used for livestock farming. We focused on behavior of farmers since they are the key decisional actors in this system as they are the ones determining land management for most of the area. Three main types of land management change affecting ES were previously identified: (1) manuring *versus* not, (2) mowing *versus* grazing, (3) early *versus* late mowing [33]–[35]. We tested the hypothesis that these three land management behaviors are driven by farmers' motivation to benefit from ES. Previous studies on farmers' behavior have stressed the need to consider multiple potential explanatory factors (e.g. biophysical, economic, political, sociological) and the relationships among them in order to address the complexity of the social-ecological system [28]. This led us to analysing the influence of multiple ES as well as a broader context of climate and socio-economic change. We built on the theoretical frameworks of Meyfroidt [5] and Vignola et al. [36] to explore the feedbacks between ES and behaviors through farmers' cognitions. First, we describe the cognitive model underpinning our analysis. We then present the methodology used to document how ES are taken into account in farmers' decisions and describe results for each component of the cognitive model. Finally, the discussion explores the feedback loop between ES and land-use through farmers' cognitive processes.

## Conceptual Framework

**Figure 1:**
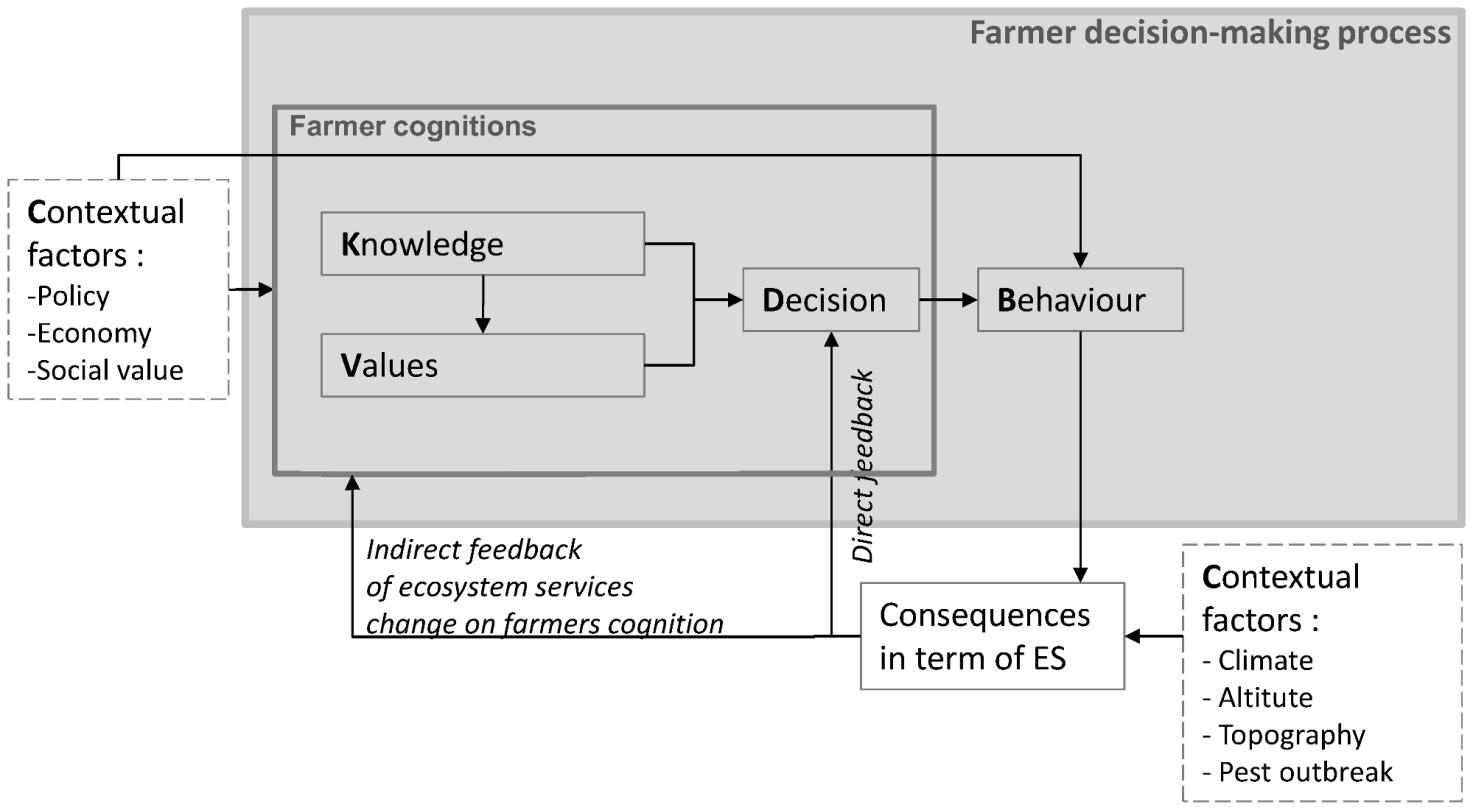
Socio-cognitive conceptual model of ecosystem services feedbacks on farmer behavior. Feedback from changes in ES supply to farmers' cognitions and behaviors can be either direct, affecting only the perceived parameters of decision, or indirect, affecting the different cognitive components underlying the behavior [5].

Land-management decisions are determined by cognitive factors regarding ES, and other contextual factors (Figure 1). Contextual factors then influence whether decisions are indeed carried on through land management behaviors.

Thus, (B  =  *f*(D, C)) and (D  =  *f*(K, V, C)), where:

- Behavior (B) refers to a series of actions (here the land-use/agricultural practices) selected among possible alternatives [28]. Behaviors follow decisions (D) except when contextual factors (C) force the agent to deviate from the preferred alternative;

- Decisions (D) refer to the preferred action selected among alternatives, taking into account the knowledge (K) and values (V) about ES, as well as the influence of contextual factors (C).

- Knowledge (K) focuses here specifically on farmers' knowledge about contributions of ecosystem functioning to ES, and on effects of their practices on these ecosystem functioning;

- Values (V) correspond to general assessments about things (here, ES) that are seen as desirable [37];

- Contextual factors (C) are factors external to farmers' cognition that can influence decisions by affecting the valence attributed to different options, or make behaviors easier or more difficult to carry out, e.g. climatic conditions, social or political context [28]. Contextual factors are also referred to in other frameworks as drivers, driving forces [38] or pressures [39]. Our focus here is on ES, which are thus presented in a separate box from contextual factors. Yet, in other studies with other objectives, ES might be considered as contextual factors themselves. We considered as ES, “the aspects of ecosystems utilized (actively or passively) to produce human well-being”. [40]. Two types of ES are distinguished here; those that can be turned directly into benefits (called ‘final ecosystem services’) and those that support other services (called ‘intermediate ecosystem service’). Before being used, consumed or enjoyed by human beneficiaries, ES should only be considered as potential ES” [1].

In the following we examine evidence for the different components of the framework and assemble them in order to understand the mechanisms of mountain farmers' decisions.

## Social-Ecological System and Methods

### a. Study area

The study site (45°03′ N, 6°24′ E, 13 km^2^) is part of the Ecrins National Park in the Central French Alps, and located on the south-facing slopes of Villar d′Arène (Figure S1a). The climate is subalpine with a strong continental influence. Mean annual rainfall is 956 mm and mean monthly temperatures range between −4.6°C in January and 11°C in July (at 2050 m a.s.l.). However, the last decade has seen several drought episodes that may be considered as warnings of future climate change. Most of the upper slopes of Villar d'Arène (above 2200 m, further called “Alpine meadows”) have been extensively grazed continuously for centuries. Since the 20^th^ century, the lower slopes (1650–2000 m), that were formerly terraced, ploughed and used for cropping (henceforth “terraces”), are cut for hay during summer or grazed during spring and autumn, and some are manured [41]. Intermediate unterraced grasslands (1800–2500 m) (henceforth “unterraced grasslands”) have been managed for hay production since the 1700s, but since the 1970's mowing has gradually ceased over 75% of the area, which is now lightly grazed in early summer (Figure S1a). Management practices are extensive, with low stocking rates and manure inputs (every two or three years), and a single annual hay cut. Trajectories of land-use changes have shaped the landscape into a mosaic of land management types resulting in distinct patterns of fertility, floristic and functional composition, and associated ecosystem functioning [42], [43].

A key element of farmers' strategy is fodder self-sufficiency. Farmers typically cannot afford to purchase the feed (i.e. hay) necessary to maintain livestock during the long winter period (6–7 months). Thus, farmers are strongly motivated to avoid purchasing feed and instead harvest and stockpile their own hay. This strategy has been challenged by recent droughts and a vole outbreak in 2009–10 which decrease fodder yield and quality. The eight farmers managing the study area can be classified in three categories according to their production: (1) 3 sheep farmers producing lambs (mean  = 21 livestock units (LU), 19 ha); (2) 3 cattle farmers breeding calves and heifers for dairy farms situated in neighbouring areas (mean  = 67 LU, 55 ha), (3) 2 farmers raising both sheep and cattle (mean  = 54 LU, 48 ha). During summer, most of the alpine meadows are grazed by a shepherd who manages local farmers' sheep along with his/her own flock (around 1400 sheep in total). The remaining alpine meadows are divided into paddocks for cattle grazing.

These farms are part of a “Less Favored Area” due to the combination of a short growing season (April-September) because of high altitude, and steep slopes (from 0 to more than 50°). Compensations for low productivity from European subsidies and agri-environmental measures to conserve mowing practices and related biodiversity constitute a significant share of farmers' income. Grasslands are collectively managed through an association called “AFP” (Association foncière pastorale) created in 1975 in which agricultural parcels of all landowners are pooled and allocated among farmers, in order to lower constraints (ex. production costs, accessibility to parcel) and increase the average size of parcels and secure land access on long-term.

In addition to agriculture, tourism is a dominant economic activity in this region recognized for its aesthetic, cultural and conservation value and recreation opportunities [44].

### b. Ethics Statement

This analysis is partially based on survey results. The interviewees were voluntary, and their answers are confidential and analyzed anonymously. Farmers surveyed are familiar with the Central French Alps Long-Term Socio-Ecological Research (LTSER) site, with whom they have been participating in research since 2003. When asking them to participate we explained that the survey contents would remain confidential and anonymous, and would not be used beyond our study. We also committed to communicate to them the results of the study, which was done in March 2013. Farmers consented verbally to these conditions, given that written consents are not enforced in the Central French Alps LTSER, nor there is an institutional review board for this study area. No specific permissions were required for research studies in this study site (45°03'N, 6°24'E). Our activities did not involve endangered or protected species.

### c. Data collection

Qualitative and quantitative data to understand the different components of the farmers' decision-making process (Figure 1 and Table 1) were collected using a role-playing game (called hereafter the “feedback game”) which took place with seven (out of the 8) farmers of the site in January 2012. The “feedback game” aimed at understanding how ES and other factors are taken into account in land management decisions in different contexts regarding levels of ecosystem service provision, socio-economic and drought conditions. The role-playing game methodology was used (i) to put farmers in an experimental situation of decisions with the help of different supports; (ii) to distinguish between what people say (‘espoused theory’) and what they do (‘theory in use’) [17] and (iii) to present to farmers how their adaptive management responding to climate and socio-political change affected ES delivery.

**Table 1:** Data collection and analysis of the different components of the conceptual model of the farmer decision-making process (Figure 1).

Objectives	Decision-making process components	Data collection	Data types	Data Analysis	Results
*Role-playing game inputs*	Contextual factors	Three socio-economic and climatic scenarios and initial boards corresponding to the three sessions of the “feedback game”	Qualitative and quantitative	“Scenarios game	
	Consequence on ES	"Feedback game” rules (number of pieces allowed in each land use type)	Qualitative and quantitative	“Scenarios game”	Table 1
*Modelling farmers cognitions*	Knowledge	"Feedback game" discussions	Qualitative	Retranscription	Figure 2; Table 6
	Values	"Feedback game" questionnaire	Quantitative	Likert-scale	Table 4; Table 6
	Decision	"Feedback game" discussions	Qualitative	Retranscription	Table 8; Table 6
	Behavior	"Feedback game" board game	Qualitative and quantitative	Digitalisation	Table 7
	Links between components	"Feedback game"	Qualitative and quantitative	Process tracing approach	Table 6
*Validation of game results*					
	Knowledge	Farmers individual + group interviews	Qualitative	Qualitative analysis of description of current practices	
	Values	Farmers individual interviews + Field function mapping	Quantitative	Anova and Chi-squared	Table 5
	Decision	Farmers individual interviews + scenarios game	Quantitative Quantitative	Regression	Table 5
	Behavior	Farmer participatory photo mapping	Quantitative	Regression	Table 5
	Contextual factors	Ancillary data	Quantitative	Regression	Table 5

The “feedback game” is a role-playing game made of a two-dimensional board game composed of cells representing a simplified landscape where farmers playing their own role were asked to place pieces representing their land management (quantity of cattle, fodder harvested and manure) according to rules translating the effects of scenarios on ES for each type of grasslands (for more information on the material see [35]). The board game retained the actual grassland types managed by each farmer, with the same proportions of grassland types, which makes it possible to compare game results with the actual land use map.

The “feedback game” was built on the outcomes of a first role-playing game (called hereafter the “scenario game”) with the same farmers (April 2011) which identified and mapped farmers' land-management adaptations to integrated climate and socio-economic change scenarios [35]. Farmers' management adaptations to alternative contexts in the “scenario game” were used to design the initial land management board of the “feedback game” and the same equipment (board and pieces) was used.

The “feedback game” was played in three sessions lasting each between 1 h and 1 h30 during the same day. Each session was composed of one round corresponding to a year where farmers were projected in a 2030-like situation according to one scenario (Table 2), in order for them to consider the effects of the adaptations they had made in the “scenario game”. These scenarios and the initial board game were used to identify the main contextual factors considered in this study (climate, socio-economic and political context from the scenarios; altitude, topography, distance to farm from the board game). The scenarios also provided a quantification of the consequences on ES delivery of land management in the “scenario game” (Table 3).

**Table 2:** Drivers and related assumptions describing the four scenarios combining climatic and socio-economic alternatives (adapted from [61]).

**Drivers**	**Climate alternatives**	
	**“Drastic”**	**“Intermittent”**
Season of drought and occurrence	Spring drought during four consecutive years	Spring or summer drought every two years
Effects on vegetation	Change in species composition. Development of species adapted to drought (eg. *Festuca paniculata, Carex sempervirens*)	No change
Effects on biomass production	Decrease by more than 50%	Decrease by 15% during drought years
Effects on water quantity (springs)	Decreased flow of all springs, even quenching of the less productive ones	Decreased flow of the springs
	**Socio-economic alternatives**	
	**“Local”**	**“International”**
Consumption demand	Local and high quality products	Cheapest prices
Aim of agricultural subsidies	To maintain both an agriculture with quality production and a high level of ecosystem services and biodiversity conservation. High subsidies but more restrictive in term of expected outcomes than in the “International” alternative.	To maintain open landscapes and production of environmental services such as carbon sequestration. Lower subsidies than on the local alternative, but less restrictive. A minimal income is guaranteed to farmers
Subsidies	20% of CAP pillar 1 support: no minimum guaranteed; Agri-environmental measures (AEM): Bonus for biodiversity with commitment to results (e.g. maintain plant diversity): 210€/ha (maximum 10000€/farm) c).; Strengthening of eco-conditionality requirements for funding (e.g. manure control)	20% of CAP pillar 1 support: subsidies generally decoupled but minimum guaranteed (1 yearly minimum wage); Agri-environmental measures (AEM): Bonus for maintaining grasslands; Carbon credits: 76€/ha (maximum 76000€/farm)

**Table 3:** Change of potential ecosystem services (decrease (↘) and increase (↗) greater than 10%) between practices in each category of grassland, for the drastic and local scenario (column “D”) and the intermittent and international scenario (column “I”) (data from [61]).

	Carbon storage		Nitrate leaching		Forage quantity		Litter quantity		Forage quality		Plant diversity		Flowering onset	
	D	I	D	I	D	I	D	I	D	I	D	I	D	I
**Manuring vs. not manuring**														
Mown terraces						↗		↘					↗	↗
Grazed terraces						↗	↗							
Mown unterraced grasslands	↘	↘					↗		↗				↗	↗
Grazed unterraced grassands														
**Grazing vs. mowing**														
Manured terraces						↘	↗	↗	↘		↘	↘	↘	↘
Not manured terraces					↘	↘	↗	↗	↘		↘	↘	↗	↗
Not manured unterraced grasslands			↗	↗	↗	↗	↗	↗	↘	↘	↘	↘		

The three scenarios used to vary the levels of ES and other contextual factors, and analyzed their effects on farmers' behaviors are: (1) the “drastic and local” scenario were repeated droughts occurring during four consecutive years with a return period of four years combined with a socio-economic context assuming demand for local products and area-related agricultural subsidies; (2) the “intermittent and international” scenario alternating favorable climatic years and droughts combined with a globalized socio-economic context; (3) the “drastic anticipation” scenario with repeated droughts as in “drastic and local” scenario, but with the current socio-economic context (Table 2). At the beginning of each session, information on ES change by land-management type (percentage increase or decrease between the current situation and 2030) was given for a set of ESs previously shown to be important for these farmers and regional experts [23]: forage quality, forage quantity, date of flowering onset, litter quantity, plant diversity, aesthetics, water quality, nitrate leaching and carbon storage (Table 3). ES changes were calculated using spatially-explicit models predicting the response of ecosystem functioning to drought and management based on plant and microbial traits, and abiotic parameters [35].

Before starting the game, each farmer ranked the value of each ES or service on a five levels Likert-scale (Table 4). This was followed by a group discussion on the attribution of values and a discussion of each service. Then, three game sessions were conducted corresponding each to one scenario. To document individual decisions in addition to collective discussions during the game, at the end of each session farmers were asked to write the reasons for adopting a given practice for each cell of the board game. The pieces placed by farmers on the board game illustrated the behaviors that they adopted. The game finished with a general debriefing where decisions and behaviors were discussed. Knowledge about ecosystem services was extracted from the discussion during the presentation of ecosystem services change before the game, through the discussions during the game and the debriefing.

**Table 4:** Ecosystem services with their values attributed by farmer (number indicates the number of farmers giving this value to a service), sorted by decreasing order of average value.

	Very low	Low	Medium	High	Very high
Forage quality				2	5
Plant diversity conservation				5	2
Forage quantity			2	3	2
Water quality (ES related to nitrate leaching EF)		1	3		3
Aesthetics	2		2	1	2
Litter quantity	2	1	2	1	1
Flowering onset	1	2	3	1	
Nitrate leaching	2	1	3	1	
Carbon storage	2	2	2	1	

Due to the complexity of the socio-ecological system, plausibility of the “feedback game” results were cross-checked and completed with information from multiple sources on actual land management behavior (Table 5). Firstly, farmers' actual land management behaviors were documented from semi-directed individual interviews conducted with the eight farmers in summer 2009 about farm structure and features of the herd, forage resource and management practices [45]. These interviews included a participatory photomapping where interviewees outlined their parcels over aerial photographs, and described the management (i.e. mowing, grazing, manuring, dates and stocking rate) as well as each parcel's “field function”, i.e. the main parcel value assigned by farmers [46]. Field functions can be interpreted as levels of ES provision and were coded according to the expectations on output: (i) both quantity and quality of fodder being expected, (ii) only quantity expected, (iii) only quality expected. We used these data to compare perceptions and actual behaviors of farmers to game results. Secondly, data about knowledge, values and decision were obtained from other surveys conducted from 2009 to 2011: (i) semi-directed individual interviews about knowledge and adaptations to past droughts [34], (ii) the ”scenario game” on adaptations to future climatic and socio-economic change under four scenarios [35], and (iii) a group interview conducted in January 2010 with 3 farmers and other inhabitants to elicit their perceptions of biodiversity and ES related to management of mountain grasslands [23].

**Table 5:** Summary of the statistical analyses at parcel level (excluding alpine meadows).

	Actual behavior (Dependent variables)			ES Values		Contextual factors					
	Manuring1a	Mowing1a	Mowing date1a	Expected forage quality1b	Expected forage quantity1b	Slope^3^	Elevation^3^	Distance to road^4^	Distance to farm^4^	Intercept	Test result
**Description**	Presence/absence of application of manure in the parcel	Mowing vs. grazing practice in the parcel	Mowing date (day) (for the year 2009)	Parcels where forage quality is expected by farmers. Quality only, or together with quantity	Parcels where forage quantity is expected by farmers. Quantity only, or together with quality	Log 10 of mean slope of the parcel (degree).	Log 10 of mean elevation of the parcel (m)	Log 10 of Euclidian distance from the middle of the parcel to the road or track suitable for vehicles (m)	Log 10 of Euclidian distance from the middle of the parcel to the farm (m)		
**Chi-square test**	X				X						X^2^ = 20,07 (1), *p*<0,001 ***
**ANOVA A**			X (early mowing)	X							F = 12,89 (2), *p*<0,001 ***
**ANOVA B**			X (late mowing)		X						F = 12,17 (1), p <0,001 ***
**Linear regression**			X				233,38*** (*p*<0,001)	6,49 (*p* = 0,06)	12,52*** (*p*<0,001)	−690,33*** (p<0,001)	Adjusted R^2^ = 0,35
**Logistic regression A**	X					−0,148** (p = 0,005)	−15,70* (p = 0,03)	0,93. (p = 0,08)	0,17 (p = 0,71)	50,59* (p = 0,03)	D^2^ (Pseudo R^2^) = 0,10
**Logistic regression B**		X				0,43*** (p<0,001)	12,51 (p = 0,20)	0,03 (p = 0,96)	−1,96** (p = 0,001)	−43,65 (p = 0,16)	D^2^ (Pseudo R^2^) = 0,42

Variables used in each analysis are depicted by “X”. The three behavioral variables (manuring, mowing and mowing date) are dependent variables, the others are explanatory variables. ANOVA and Chi-square tests discriminate pairs of variables depicted by “X”. Regression results presented for each variables are parameter estimates and p-value. Significance levels:

* (0.05);

** (0.01);

*** (0.001), N = 217 parcels.

Data sources:

1a Land managements and ^1b^ field functions from participatory photo mapping;

2 Digital elevation model;

3 Land use map;

4 ArcGIS Euclidian distance based on Land use and topographic maps.

Finally, ancillary spatial data was used to study the effects of contextual factors (i.e. altitude, topography, distances), on land management behaviors: a land-use map of the site constructed using a combination of cadastral (1810 to 2009) and aerial photographic data (since 1952) [41], a 10 m×10 m Digital Elevation Model, and settlements, farms and road digitized from the 1∶25000 topographic map (IGN).

### d. Data analysis

**Figure 2:**
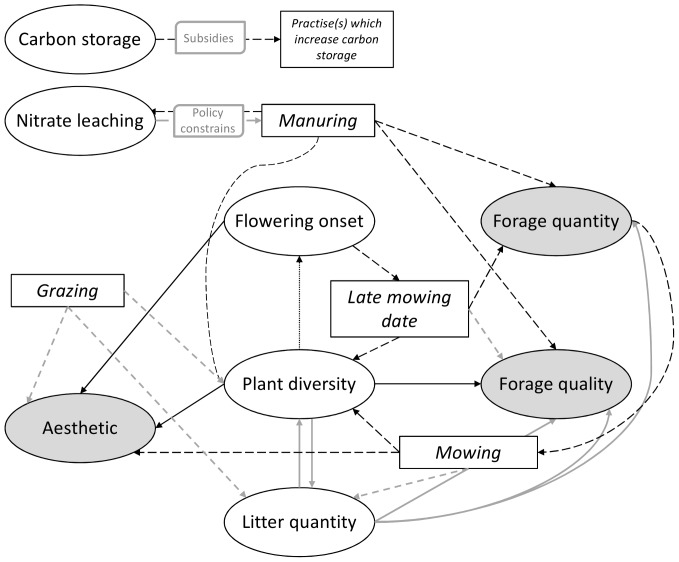
Farmers' ecosystem services values and knowledge. Conceptual representation based on farmers' discourses on values and knowledge about the relationship between ES and land-management practices. Rectangle boxes indicate practices and ellipses indicate ES. Dashed arrows indicate links between practices and ES and plain arrows indicate links between ES. Grey arrows indicate a negative effect and black arrows a positive effect. Except for the effect of litter quantity on forage quantity, farmers agree on all the relationships. Note that ES in grey are seen as final ES by farmers while the others are considered as intermediate ES [40].

Interviews and game discussions were recorded, typed and coded by themes using Nvivo 9 (QSR International) to extract the different components of farmers' cognitions (Table 6; Figure 1: values, knowledge, decisions) for each ES (Table 4) and draw the mental map (Figure 2). The “feedback game” questionnaires about values were analyzed using the Likert-scale data (Table 4). Land management type and their location on the boards of the “feedback game” sessions were recorded (photography and GIS) and analysed for each farmer to collect data on behavior (Table 7).

**Table 6:** Representative quotes extracted mainly from farmers' discussions and the debriefing of the “feedback game” (7 farmers, January 2012).

	**Knowledge**
	**Quotes about relations between ecosystem services:**
1.1	“A *beautiful grassland with a lot of flowers, it's more beautiful than a grassland with only Festuca paniculata”*
1.2	“*diversity corresponds to quality*”
1.3	“*after one year, we can see the effect during the spring on plant re-growth on grassland which are grazed only a little bit. It's protected from frost”*
1.4	“*in a grassland that I have not grazed a lot, in autumn and even next spring nothing regrows*”
	**Quotes about relations between ecosystem services and practices:**
2.1	“*We manure only with natural fertilizer* (manure). *It is not certified organic, but we do not use mineral fertilizer, so we are far from this kind of problem*”
2.2	“*Rain or snow seep manure into the soil. There is no leaching*”
2.3	“*I take that into account because I have a plan for spreading manure agreed with the authority”*
2.4	“*Today, everything shows me that fertilizing increases forage quantity*”
2.5	“*Do not manure beyond some limits, because after you change the flora*”
2.6	“*In the autumn manure rots better than during spring when it's dry. After we have it on fodder*"
2.7	“*Farmers are all aware that the floristic diversity will change if we stop mowing*”
2.8	“*It* (mowing) *maintains an open landscape*”
2.9	“*We wait as long as possible until plants are at flowering stage. We maximize quantity. But that's not the best* (for quality)*”*
2.10	“*Mowing too early, before July the 20^th^, doesn't leave plants time to set seed and then decreases the number of species*”
2.11	“*The sheep do not put their head into* (Festuca paniculata), *but cows manage to pull out a few*”
2.12	*“The quality of fodder is linked to farmer's work. The way the grassland is managed every day”*
	**Quotes about the effects of additional factors on ecosystem services**
3.1	“*In 1988, rain occurred throughout June, there was so much fodder that we could not give it all, we wasted a lot because it was hard, coarse and the sheep didn't want to eat it*”
3.2	“*Summer rains lead to a bit of second growth*”
3.3	“*With this spring drought we did not have a lot of forage*”
3.4	“*If vegetation starts to grow too late, at 2000 meters of altitude if a cold snap occurs, the vegetation does not restart*”
3.5	“*Snow is also needed to rot plant litter*”
	**Values**
	**Quotes about values of ecosystem services**
*4.1*	*“There is a difference between fodder, and a palatable fodder consumed by cows”*
*4.2*	*“That's nice to have fodder in quantity but if it's crap fodder … you have only crap fodder”*
*4.3*	*“It's the balance between quality and quantity that is interesting”*
*4.4*	*“If we do not have quality fodder, we will have to buy quality fodder to compensate”* (all farmers)
*4.5*	*“In the cost of one hectare of mowing grassland, there is also the result in terms of forage quantity and quality to take into account”*
	**Behaviors and explanations**
	**Quotes about land management practices**
5.1	“*Our herd is our business, therefore we keep our herd and we adapt the rest* (the area mowed) *on the herd”*
*5.2*	*“We will not manure if this does not bring quantity”*
*5.3*	*“I am perturbed. This grassland in altitude* (unterraced grasslands) *should stay mown. It's better to have a spread in fodder than have fodder at a single altitude”*
	**Quotes about changes in environmental cognitions (knowledge and values) arising as indirect feedbacks from changes in ecosystem services**
*6.1*	*“During years of crisis, we look first at quantity and quality, before considering colours of flowers and all these things. If you asked us the same question some years ago, we would probably not have answered the same thing”*
*6.2*	*“Some years ago, I was more or less independent for fodder. I was looking mainly for quality to have a specific fodder for lambs and calves”*
*6.3*	*“Due to the vole outbreak, we had bad fodder because soil was collected along with fodder. This led us to think differently”*
	**Quotes about contextual factors affecting the decisions and alternative hypotheses**
*7.1*	*“We manure the best and flattest parcels”. “Manured and grazed … how is it possible? Only* (one farmer) *has flat land… anyway”*
*7.2*	*“We will not manure where there is drinking water extraction”*
*7.3*	*“I do not take into account distance to stream, because we have a lot of streams and with 30 meters we are far into the parcel”*
*7.4*	*“In the lower part, I have to continue to fertilize, because I have to empty the manure pit …”*
*7.5*	*“In this parcel we cannot load the hay. We need to bring it down to the road”*
*7.6*	*“Here, mowing currently grazed parcels is not possible. There is no lands were a return to mowing is possible”*
	**Behaviors and explanations**
	**Quotes about contextual factors affecting the decisions and alternative hypotheses**
*7.7*	*“The problem with grazing is that we need water supply. A cow consumes 40 liters per day and there is not always an access to carry water”*
*78*	*“We will try to continue to mow as far as we can by respect towards elderly people … but on mechanisable parcels”*
*7.9*	*“To respect their work, the terraces they built”*
*7.10*	*“We have to respect land. Not entering when it's wet, and not grazing when mowing is possible. When a terrace is grazed it's due to an accessibility issue”*
*7.11*	*“Grazing instead of mowing is another system; the agreement of the landowner would be needed”.*
*7.12*	*“What we will do during summer if we stop mowing? We will have a lot of time. We are not shepherds”*
*7.13*	*“If land becomes available, I will stop to mow over the entire landscape and do it near my farm, to waste as little time as possible. Even if I will need to increase by two the hours per day or to take an additional worker during a few days”*

Quotes illustrate the different components of the conceptual model presented in Figure 1.

**Table 7:** Farmers' behaviors in reality and in each scenario (“feedback game” session) for each type of grasslands: Mown terraces; Grazed terraces, Mown unterraced grassland, Grazed unterraced grasslands.

	Manuring				Mowing (vs. Grazing)			
	Mown terraces	Grazed terraces	Mown unterraced grassland	Grazed unterraced grasslands	Mown terraces	Grazed terraces	Mown unterraced grassland	Grazed unterraced grasslands
**Current practices (2009 data)**	Y/N	Y/N	Y/N	Y/N	Y	N	Y	Y/N
**Scenario “drastic and local”**	-	Y/N	N	N	-	N	N	N
**Scenario “intermittent and international”**	Y	Y/N	Y/N	Y/N	Y	Y/N	Y/N	N
**Scenario “drastic anticipation”**	Y/N	N	Y	Y/N	Y	N	Y	Y/N

“–“ means: no information. “Y” means they adopted the behavior and “N” they didn't. Y/N indicates that both behaviors were adopted by farmers of the area.

Maps resulting from participatory photomapping were digitized and georeferenced (with ARCGIS, ESRI), to overlay with the other maps. In order to test whether relations between behaviors and other elements discussed during the game process were consistent with those in the real life, we performed statistical analyses (ANOVA and regressions) of the relationships between actual land management behaviors (mowing, grazing or manuring), as dependent variables, and ES (expected values in term of quality and quantity identified by farmers (field functions)) or potential contextual factors (listed in Table 5) as explanatory variables (Table 5) using R (R Development Core Team 2008). These statistical analyses provide one additional element to test the main hypothesis, i.e. the effect of ES knowledge and values on farmers' decisions.

The entire feedback loop from change in ES supply to farmers' behaviors was then analyzed by combining all this data and using the « process-tracing » approach [47] to explore individually each component of the conceptual model before considering links between them (Figure 1) (as in [31]). This method attempts to identify the causal chain and mechanisms between independent variables (cognitive factors such as knowledge, values and decisions, and contextual factors such as climate change or the socio-economic context) and the outcome of the dependent variable (farmers' behaviors). Tracing all the steps in the process chain linking knowledge and values to behavior and the consequences in term of ES – Figure 1), and all the necessary implications of the main hypothesis (farmers' land management behaviors are driven by their willingness to benefit from ES) provides evidence to test this main hypothesis. Meanwhile, the alternative paths through which the same outcome could have occurred, without being influenced by perception of ES (e. g. through effects of external factors on behavior) were identified and tested, also by being decomposed as a series of steps [47].

## Results

This section presents successively the different components of the decision-making framework: (a) cognitive variables (knowledge and values) about ES and practices, (b) behaviors and their explanations provided by farmers (decisions) and influences of environmental cognitions and ES on farmers' land management behaviors. Finally, we explore (c) factors other than ES which influenced farmers' behavior.

### a. Farmers' environmental cognitions

#### Knowledge

This section describes farmers' understanding and perceptions of: (1) each ES and its relationships to others, (2) relationships between ES and agricultural practices (Figure 2), and (3) effects of contextual factors on ES.

The ES described by researchers (see Figure 2 for the list of ES) were all known by farmers except nitrate leaching and carbon storage which required more explanations. Farmers were knowledgeable about ES, even without calling them “ecosystem services” [23], [34]. Several types of influences among services were recognized: (i) services mutually influencing each other (e.g. plant diversity, flowering onset and litter quantity), and (ii) some ES are only influenced by other services but do not themselves have influence on others (aesthetic, forage quantity, forage quality) (Table 6, quotes 1.1 to 1.4). Finally, some relationships, e.g. between nitrate leaching or carbon storage and other services were not mentioned, even after our explanations.

Regarding the mutual influences between ES and practices, farmers did not perceive manuring to affect nitrate leaching in their agricultural system (Table 6, quotes 2.1 and 2.2). Nevertheless some farmers were mindful of nitrate leaching because of legislation. Farmers considered that manuring unterraced grasslands may increase forage quantity and quality, and also plant diversity, but did not wish to apply manure more often or in greater quantity than under current conditions (Table 6, quotes 2.4 and 2.5). Spreading manure in autumn was considered more efficient compared to spring, and soiling fodder was avoided (Table 6, quotes 2.1 and 2.2). Mowing was considered to increase plant diversity but also directly aesthetics (Table 6, quotes 2.7 and 2.8). Moreover, farmers asserted that the decision to mow was influenced by productivity in a given year: some of them do not mow when forage quantity is not considered worthwhile. The timing of mowing influenced the forage quality (expected to decrease with late mowing date) and quantity (expected to increase with late mowing date) of forage harvests, leading to a trade-off between both services. But a late mowing date was also perceived to increase plant diversity, thus indirectly forage quality on long-term. Mowing date was in part motivated by the date of flowering onset of dominant grasses. Farmers agreed that lower parcels (terraces) are mown at the beginning of July, and higher parcels not before the 10^th^ August in years with early vegetation onset, and some years not even before the 20^th^ August (Table 6, quotes 2.9 to 2.12). Finally, ES influencing the decision to shift to grazing were not mentioned, but they usually grazed if there is no enough forage quantity to mown. Grazing was mentioned to have negative effects on aesthetics and plant diversity, and to decrease litter quantity, though less than mowing.

The effects of additional factors, such as climate, altitude or a recent vole outbreak on ES and practices were also discussed. Rainfall influenced forage quality and quantity (Table 6, quotes 3.1 to 3.5). Forage quantity was also known to be influenced by temperature and altitude. Altitude was perceived as influencing the date of flowering onset more than plant diversity. The presence of snow was considered to affect litter decomposition. The effects of these external factors on practices will be presented in the “alternative hypotheses” section (see also the alternative hypothesis section of Table 6 and the contextual factors section of Table 8).

**Table 8:** Factors influencing farmers' decisions to adopt a practice during the “feedback game”, according to farmers accounts and discussions.

	Manuring	Mowing	Date of mowing
***Ecosystem services***			
**Forage quality**	X	X	
**Plant diversity conservation**	X	X	
**Forage quantity**	X		
**Litter quantity**		X	
**Flowering onset**			X
**Nitrate leaching**			
**Carbon storage**	X	X	
***Contextual factors***			
**Steep slope**	−	−	
**Altitude**			+
**Proximity to farm**	+	−	−
**Low accessibility**		−	+
**Proximity of dwellings or streams**	−	−	
**Parcels surroundings**	−	−	−/+
**Availability in manure**	+		
**Equipment costs**	−	−	
**Social conflicts**		+	
**Social value of farming**		+	+
**Subsidies amount**		+	+
**Policy or legislation constraints**	−	−	+
**Availability of land**		+	
**Snow or rain**	−		+

The first part of the table presents ecosystem services, with a X when a given service is said to influence a given practice (manuring, mowing, late mowing). The second part presents other contextual factors, with their positive (+) or negative (−) influence on the decision to adopt a behavior corresponding to alternative hypotheses.

#### Values

By averaging the scores of importance given in ranking tables filled individually, ES were ranked by decreasing value and desirability (Table 4). Higher values were attributed to final ES from which farmers benefit directly [1], including forage quality and quantity, while intermediate services to the production of final ES, except plant diversity, received lower values. Farmers consistently attributed high values to some services like plant diversity, forage quality and forage quantity, while there were more heterogeneity in values attributed to other services.

The reasons for these rankings were then expressed by farmers during a collective discussion following the Likert-scale rating exercise before the “feedback game”. Forage quality was considered as highly desirable for herd welfare or for parts of the herd with higher needs such as lambs or dairy cows, and for some farmers was complementary to forage quantity (Table 6, quotes 4.1 to 4.5). At the same time, forage quality and quantity were also factors contributing to farm economy. In addition to the information on value, Figure S1b shows the location of parcels where quality and/or quantity were expected according to the field functions mapped by farmers (photomapping interviews). Plant diversity was also highly valued by farmers for its contribution to forage quality, to aesthetics, or both, consistent with the indirect links suggested between these functions (Figure 2). Litter quantity received very varying values, considering on one hand a positive short-term effect on vegetation re-growth due to protection against frost and a fertilizing effect of litter when mown every couple of years, and on the other hand a negative long-term effect as litter chokes out vegetation and then decreases forage quantity and quality. Some farmers considered only the negative or positive effects of litter quantity. Low scores of carbon storage were probably due to a lack of knowledge rather than lack of interest. Nitrate leaching received a low value, probably because farmers did not feel concerned by nitrate leaching, or because it was seen as having a negative influence on water quality which was generally highly valued.

### b. Farmers' behaviors and explanations

For each practice, this section describes, (1) behaviors adopted by farmers during each “feedback game” session based on board game analyses (Table 7); (2) actual farmers' behaviors reported on maps of actual practices during the 2009 participatory photomapping (Table 7 and Figure S1); (3) the explanations given by farmers during the “feedback game” of the influences of ES on their behaviors (Table 6 and Table 8); and finally (4) validation of farmers' behaviors during the “feedback game” by analysing the relationships between actual land management behaviors and field functions interpreted as ES values (ANOVA Table 5).

#### Manuring vs. non manuring

In the “drastic and local” scenario most farmers stopped fertilizing terraces and unterraced grasslands. By contrast, during the “intermittent and international” scenario, they all increased the number of terraces manured, except one who stopped manuring them. Finally, in the “drastic anticipation” scenario, two farmers stopped manuring terraces, one of them started to manure mown grasslands, and two others manured grazed unterraced grasslands.

In the actual practices in 2009, farmers did not manure all their land, but only some mown terraces (Figure S1c). Grazed unterraced grasslands were not manured. Sheep farmers did not manure at all and farmers having both sheep and cattle used only cattle manure (sheep manure is given to a compost making firm).

Farmers did not manure when this did not maintain or increase forage quantity (Table 6, quote 5.2), forage quality or plant diversity (as in the “drastic-local” scenario), while they manured when it did (as in the “intermittent-international” scenario). Manuring of parcels to increase forage quality coincided with the desire to increase forage quantity both in reality and in the “feedback game” sessions. One farmer manured terraces and another one unterraced grasslands to increase carbon storage and hence receive carbon credits as proposed in the “intermittent-international” scenario. Nitrate leaching was never mentioned as a reason to adapt manure practices.

The comparison between the maps of actual practices (Figure S1c) and of expected quantity and quality of fodder (Figure S1b) showed that manure was applied mostly on parcels where changes in forage quantity were expected more than changes in forage quality (Table 5, chi-squared test).

#### Mowing vs. grazing

In the “Drastic and local” scenario, terraces were mainly grazed at the expense of mowing. Half of the farmers stopped mowing unterraced grasslands, in order to feed herds during grazing seasons. Then they had to buy fodder for winter. During the “Intermittent and international” scenario, terraces were mown, and mowing was resumed on some grazed terraces. Unterraced grasslands were mainly grazed. Only two farmers manured some unterraced grasslands. In “drastic anticipation”, one farmer continued mowing them and another farmer even mowed and manured previously grazed unterraced grasslands.

In the current practices, farmers organized their land management around spring grazing and mowing, because summer alpine meadows are large enough to ensure flexibility in forage resources (Figure S1c). During autumn, the herd grazed extensively on the re-growth of mown or spring grazed grassland. Areas of grazed versus mown unterraced grasslands were adjusted to herd size (Table 6, quote 5.1), while the remaining area was used to mow, leading three farmers to harvest part of their fodder in other municipalities.

Some farmers attributed their decision in the RPG to mow terraces to its positive effect on forage quality and on the reduction of litter (in the “drastic-local” scenario), or to its benefits for plant diversity and forage quality (in the “intermittent-international” scenario). Date of flowering was also cited once as a factor influencing decision to mow terraces. Unterraced grasslands were also mown by some farmers to increase or maintain plant diversity and decrease litter quantity, in the “drastic-local” scenario. By contrast, these grasslands were grazed to increase carbon storage in the “intermittent-international” scenario.

Maps of expected ES (field functions) showed that in mown parcels farmers often expected to obtain high fodder quality as well as large fodder quantity (Figure S1b). Mown parcels where quality was expected were concentrated on the lower part of the slope, mixed with parcels where only quantity was required.

#### Date of mowing

Dates of mowing were not discussed during “feedback game” sessions. During interviews and participatory photo mapping, farmers explained that dates of mowing are spread between 1^st^ July and mid-September (FigureS1d).

One farmer indicated that, by choosing to graze unterraced grasslands to increase carbon storage (Table 6) and gain credits as proposed in the “intermittent and international” scenario (Table 2), dates of mowing on his parcels were disturbed as he had lost the possibility of later mowing in unterraced grasslands (Table 6, quote 5.3). This farmer faced a trade-off between maintaining a spread in mowing date or receiving carbon credits and continued to graze according to the “intermittent and international” scenario.

Map comparison revealed associations between early mowing (current practice) and expected quality or late mowing and expected quantity (Table 5, ANOVA) (Figure S1b and S1d). However there were no significant relationships between date of mowing and actual date of flowering onset, or plant diversity (Simpson index) and date of mowing.

### c. Contextual factors affecting the decisions and alternative hypotheses

This section describes contextual factors (internal or external to the farm) influencing farmers' decisions, using explanations by farmers (during games or interviews) and statistical analyses between spatial factors and practices (Table 5). These factors can explain divergences between farmers' behaviors and attitudes, or in case of consistent behaviors, constitute alternatives to our main hypothesis that farmers' land management behaviors are driven by their willingness to benefit from ES.

#### Manuring vs. non manuring

As mowing and manuring are mechanised, constraints on the mechanisation of parcels such as slope and accessibility came out as a recurrent theme in farmers' discussions (Table 6, quote 6.1). Distance to farm was mentioned as a factor influencing manuring due to price of fuel and travel time to the parcel (except by one farmer who rents a truck bringing manure close to the most remote parcels). Other characteristics of parcels were sometimes considered such as proximity to dwellings, streams and water springs (Table 6, quote 6.2 and 6.3). These aspects were mainly considered because of legislation and policy support including fertilisation management plans, which impose quantity, date and distance thresholds. Sheep farmers usually did not use their manure and gave it to a specialized company, which does not take liquid manure or slurry. Therefore, the capacity of slurry storage pits forced farmers to manure when it was full ((Table 5, quote 6.3). Individual parcels were manured only once every two or three years. Finally, for some parcels, the short time between autumn grazing and snow (around 1^st^ November on average) limited manuring. Mineral fertilisation was considered as too costly.

The effects of some contextual factors mentioned by farmers were confirmed by statistical analyses on land use maps. The logistic regression of factors influencing manuring showed that manuring was mainly applied on gentle slopes, but distance to farm, distance to road and altitude were not significant (Table 5, logistic regression A). In addition, we estimated the maximum area which could be manured according to the amount of manure produced depending on farm characteristics. This theoretical calculation considered farm herd size, an average annual production of manure of 4.5 T per livestock unit, a theoretical average of 15 T/ha of manure per spreading and a frequency of manuring of every two or three years for each parcel. The results suggest that all farmers could potentially fertilize almost all their mown grasslands at the selected frequency, or that they could increase frequency of manuring on usually fertilized parcels.

#### Mowing vs. grazing

Mechanisation of parcels was mentioned as an important determinant of conversion from mowing to grazing (Table 6, quote 6.5). At the time of data collection farmers considered that they were mowing all the mechanisable parcels (Table 6, quote 6.6). They regarded mowing equipment adapted to mountain terrain as too expensive. Factors related to farms' economy, such as cost of mowing considering investment into equipment (purchase cost, depreciation and maintenance), appeared often in farmers' discourses. This was even more prominent when the farmer had acquired new equipment to meet the norms of the European Union and/or when farm debt level was important. When asked to rate the importance of equipment investments compared to ES in their behavior, farmers responded “very high” for cost of mowing and “high” for ES. Some equipment such as manure spreaders was shared between farmers to decrease costs, but each farmer usually owned their personal cutting equipment whose use could not be spread through time. Agri-environmental measures provided subsidies to mow unterraced grasslands and a possibility of extra subsidy to mow less mechanisable parcels with a pedestrian mower. While this kind of subsidies substantially contributed to the farms' economy, farmers argued several times during the “feedback game” that, although policy supports were carefully taken into consideration (balancing the financial amount and constraints), their amount were insufficient to compensate the actual cost of maintaining mowing practices.

Farmers discussed different elements favouring conversion from mowing to grazing. They explained that mown parcels surrounded by grazed parcels belonging to other farmers could be more prone to conversion, to avoid risks like trampling by cattle or fence removal. The altitude of the site did not allow multiple uses of parcels throughout the season (e.g. grazing before mowing) because vegetation re-grew only at the end of summer, and in small quantities. Parcels close to the farm were needed to turn out the herd to grass during the first weeks of spring. Grazing also required the presence of water or the possibility to install a trough (Table 6, quote 6.7). Future opportunities to mow elsewhere or to acquire parcels from retiring farmers might arise, allowing remaining farms to increase their land and then fodder production, or to restrict mowing to the more mechanisable parcels and to graze the others. By consolidating land among farmers, the “Association foncière pastorale” has allowed them to increase the average size of their parcels and decrease their production costs, but also to solve conflicts between families about some parcels, allowing to manage them effectively. This could in turn favour mowing because some farmers perceived a social pressure to properly manage land, and thus to prevent shrub encroachment, especially in terraces, which have a high cultural value and suffer from trampling by stock. This is directly linked with farmers' perceptions of the social value of farming and of social pressure from landowners, other farmers and/or inhabitants (Table 6, quote 6.8 to 6.11). Mowing also appeared as an important aspect of the farming profession: in the discussions, the possibility of completely stopping mowing was always a source of laughter (Table 6, quote 6.12).

The logistic regression on mapped data confirmed farmers' explanations that mowing, in contrast to grazing, was preferentially located on parcels with gentler slopes and further from farms. Altitude and distance to roads were not significant in the model (Table 5, logistic regression B).

#### Early vs. late mowing

For farmers having contracted agri-environmental measures, mowing cannot occur before the 1^st^ July on unterraced grasslands. This subsidy was perceived as a constraint by some farmers, depending on the inter-annual variability in flowering date. Having parcels spread across the landscape increased the time necessary to mow them all (although re-parcelling between farmers decreased that constraint), but the altitudinal span of parcels allowed them to stagger the mowing over the summer season. This was seen by some farmers as an opportunity, while others perceived it more as a constraint and argued that ideally it would be preferable to have the entire mown area around their farms (Table 6, quote 6.13). All farmers worked alone on their farm, and additional labour was hired exclusively from family when needed, e.g. for mowing. On parcels enclosed within other farmers' parcels, farmers of the enclosed parcel had to wait for surrounding parcels to be mown first, before being able to harvest their own parcel. Around the villages and campsites, some parcels were mown earlier than desired to avoid trampling by tourists. Finally, mowing dates depended on rain as dry weather is necessary to harvest fodder.

According to spatial data, early mowing occurred mainly on the lower part of the area and late mowing on the upper part, except for some parcels (Figure S1d). A linear regression model confirmed that not only parcel elevation but also distance to farm had an influence on the date of mowing (Table 5, linear regression).

## Discussion

First, results are discussed looking at how knowledge and values about ES influence behavior, and how contextual factors can change farmers' cognitions or decisions. Second, methodological relevance is discussed. Third, implications of findings for future studies on ES are examined.

### a. Role of ES in farmers' decision-making process

Visions of ES differed between farmers and scientists [48]. Farmers explained that for them, *“ES are neither numbers nor upward or downward trends*”, but are part of a more complex system of decision-making. Here we discuss the correspondence between farmers' willingness to adopt different behaviors according to their values and knowledge towards ES, and farmers' behaviors in the “feedback game” or actual life. Returning to the conceptual chain (Figure 1), three configurations emerge, explaining whether ES were taken into account in farmers decisions or not. First, some ES were not part of farmers' knowledge or far away from their interest and therefore had low values. This was the case for carbon storage and nitrate leaching, which were thus in principle not considered by farmers when making decisions. Yet, institutional mechanisms may lead some farmers to consider these services [49], as demonstrated in the second “feedback game” session where contractual carbon credits could be allocated to farmers, which indeed modified some decisions. Second, some ES were known by farmers but had a low value, or farmers considered not having enough knowledge to include this ES in their decisions. For example, farmers perceived an influence of mowing date on the date of flowering onset, itself affecting forage quantity and quality, but they did not consider themselves capable of obtaining a desirable ecosystem service delivery by adjusting mowing dates. Additional reasons (section 3.3, e.g. distance to farm, surroundings of the parcels, weather) also constrained mowing dates. Third, in some cases farmers had both knowledge and high values for given ES. “Feedback game” sessions showed that more parcels were manured when it enabled increased forage quantity and secondarily forage quality or plant diversity. According to their knowledge late mowing could be favored to improve forage quantity, as well as plant diversity and thus indirectly forage quality on long-term. But, because late mowing also decreased forage quality directly, trade-offs had to be considered. In this case, farmers could opt for the most highly valued ES and adapt their behavior accordingly. Whatever the behavior adopted, in this case farmers took multiple ES into account in their decision. These results suggest that knowledge and/or values were necessary but not always decisive in farmers' decisions.

Results also suggest that both direct and indirect feedbacks (Figure 1) explain how ES were taken into account in farmers decisions. Most changes in ES during the “feedback game” had direct feedback effects on farmers' decisions because these represented changes that farmers face frequently (e.g. change in fodder quality or quantity due to weather conditions). But an indirect feedback was also observed with the case of carbon storage. Farmers were not aware of carbon storage before the “feedback game”, and some changed their values and knowledge about it, so that for some this factor entered in their decisions. This was also the case for their knowledge about the effects of manuring on forage quantity during drastic drought. While in the previous “scenario game” analysing farmers' adaptation to climate change, farmers increased manuring to face droughts [35], in the first session of the “feedback game” some farmers considered the results presented to them on ES impacts (Table 3) and, realizing the inefficiency of this practice under drought, stopped manuring. Occurrence and amount of change in ES could also influence the feedback type. Short-term or small changes in ES affected farmers' behaviors mainly through direct feedbacks leading to tactical adaptation (e.g. conversion of mowing to grazing on a given year). By contrast, greater or frequent changes in ES supply (e.g. repeated drought decreasing forage quantity, or a vole outbreak during several years) could lead to change in values (Table 6, quotes 7.1 to 7.3).

Nevertheless, it would be naive to consider that ES fully drive farmers' behaviors. Indeed, behaviors did not always correspond to their attitudes regarding ES and this could depend on the parcels considered (section 4c). Other factors have been shown to influence land use practices in European mountain systems: parcel characteristics (e.g. topography, location, size, land-locked position, proximity of water supply), market prices (e.g. input prices and output prices of production), policy support (e.g. types and amount of subsidies), climate (e.g. drought, frost, rain) and pest outbreaks (e.g. voles, grasshoppers) [27], [50], [51]. Social factors also matter, including structure of the farm business (e.g. farm type, farm size, farm economy), farmer characteristics (e.g. age, gender, education, and personality), household characteristics (e.g. level of pluri-activity, work pattern of the spouse), and structure of the social environment (e.g. local culture, social capital, information flows) [52]. On the one hand, our results confirmed that, although the influence of ES is not negligible, in some cases these other factors outweigh ES in farmers' decisions. For example, the difference in manuring between repeated and intermittent drought conditions (“feedback game” sessions on “drastic and local” and intermittent and international” scenarios) confirmed that forage quantity influenced farmers' decisions, because they fertilised only when it was efficient. Nevertheless, some parcels were not manured (in “feedback game” sessions on “intermittent and international” and “drastic and local” scenarios) because some of them were not mechanisable, too far from the farm resulting in high transport cost, or at unauthorised distance from streams or settlements imposed by regulations. By contrast, despite the inefficiency of manuring in repeated drought conditions, some farmers still manured some parcels because they had to use their manure. During the “feedback game” farmers chose to mow some parcels to increase forage quantity and/or quality, plant diversity or decrease litter quantity but sometimes grazing was preferred due to the cost of mowing, accessibility and possibility to mechanise. Financial incentives also had importance in farmers' decision as shown by the farmer grazing a parcel to receive carbon storage subsidies although mowing would have been preferred for forage quality. Farmers took into account date of mowing to increase forage quantity or quality, but some parcels around villages were mown earlier than expected to avoid trampling. Similarly, unterraced grasslands were mown too late because of the time needed to mow the other parcels nearer to the farm. On the other hand, even when behaviors were found to be consistent with attitudes towards ES, alternative reasons could also have driven farmers to adopt these behaviors. ES would thus be only one factor among others contributing to farmers' decisions. For example, some farmers mowed parcels to increase plant diversity, but this could also have been favored by financial support from agri-environmental measures and social value attributed to mowing as part of the farming profession. Agri-environmental measures could also favor late mowing, as they impose a date threshold.

### b. Methodological relevance

The results presented are valid for our study located in a high mountain farmer community of the French Alps, where agriculture is very extensive. Although to our knowledge no previous study has analyzed the complete feedback loop, some studies suggested that farmers in other contexts have detailed and accurate knowledge about the relationship between their practices and the functioning of their agro-ecosystem [27], [51]. Therefore, our results might hold for other mountain agricultural social-ecological systems where farmers opportunities (policy support, economy) and constraints (e.g. topography, weather conditions, higher cost of productions) to adopt different behaviors are to a large extent similar [50], [51].

The results of this study are based on data gathered from eight farmers which represent the total farming population of the area. This small number did not allow statistical analyses on decision-making following established paradigms such as the Theory of Planned Behavior (TPB) or the Value-Belief-Norm Theory (VBN) [53] but enabled deeper analysis of the reasons behind the relationships between the different components of farmers' decision-making process and of their effects on behavior. The direct involvement of farmers during the role-playing game and their adaptations in a realistic situation of change allowed us to observe and to discuss how they decided to adopt a given behavior [35]. The coherence of results was increased by cross-checking data from different sources of information. This led us to use a combination of qualitative and quantitative methods which have both strengths and weaknesses.

Spatial analyses (photomapping of actual behaviors) provide robust quantitative data on decisions, but do not give information to interpret them in terms of ES values and knowledge. Interviews provide useful qualitative data to interpret the reason behind decisions, but give little quantitative information to assess whether rationales given by the farmers are effectively implemented. Role-playing game provide a crucial link between quantitative spatial data and qualitative data from interviews, by providing explicit, quantitative data on decisions, though less robust than spatial analyses of actual behaviors, and less self-explanatory than interviews.

Participatory simulation, of which role-playing game constitute one tool, is a very useful approach in decision-making processes in complex systems [54], [55]. However, this approach implies different forms of learning such as the learning related to the stakes involved, to the other players or to the technical aspects [55]. The addition of a protocol to assess farmers' learning as an outcome of the iterative process of our approach could be beneficial.

### c. Implications for ES research

Our results show the importance of considering stakeholders' perception and use of ecosystems rather than focusing only on potential ES supply [56]. Stakeholders' perceptions and potential ES delivery sometime differ [1] and the latter may not coincide with stakeholders' needs. Nevertheless, most of the potential ES presented to farmers were indeed considered by them as services, except for water quality/nitrate leaching and carbon storage of which they were not aware. Studying systemic representation of potential ES by farmers (Figure 2) also allowed us to show that potential ES can benefit to farmers (i) either individually (forage quality, forage quantity, litter quantity, date of flowering onset, plant diversity and aesthetics) or as tradeoffs (forage quantity and quality), and (ii) directly (i.e. final ES: forage quantity, forage quality, aesthetics) and/or indirectly (i.e. intermediate ES: flowering onset, plant diversity, litter quantity) [40]. For example, plant diversity was not considered by farmers for its intrinsic value but rather for its value to contribute to forage quality and aesthetics. Quality and quantity of “green” forage (vegetation) were seen as services deriving directly from the ecosystem, while quality and quantity of “dry” fodder (harvested) were considered as benefits, acknowledging that manufactured and/or human capital (e.g. farmer know-how) are required to generate a valued good from ES (Table 6, quote 2.12) [1], [6]. Moreover, our results showed the importance to not only consider perception or valuation of ES by stakeholders but also their effective uses. ES potentially supplied, ES perceptions and ES actually used can differ according to individuals and to the spatial and temporal contexts. For example farmers might not manage a parcel with high plant diversity towards quality fodder because of topography limiting access, or because of the context forcing them to maximise quantity at the expense of quality. For the purpose of ES conservation, it is also important to consider farmers' awareness, willingness and/or ability to adopt a practice maintaining or enhancing ES delivery in the social-ecological system as a whole. Results of this study confirmed and illustrated the utility of decomposing ES along the conceptual cascade from ecosystem processes to values [1], [57], [58].

Although all components of agro-ecosystems cannot be translated in terms of ES, research focusing on ES can complement agronomic studies for several reasons. Firstly, the ES framework translates ecological complexity into common language easily understandable by researchers from different disciplines as well as farmers, stakeholders or policy makers [59]. This study showed that the concept was rapidly understood, even by farmers who had never heard the term before [23]. Secondly, it emphasizes human-environment interactions which have been generally overlooked in previous research, increasing the awareness of the dependence of society on biodiversity and ecosystems [60]. Thirdly, it allows identifying and arbitrating trade-offs and priorities at the farm, municipality or even larger scales involving beneficiaries having different interests. In this study, the national park aims at maintaining mowing in unterraced grasslands to conserve this rare agro-ecosystem and the related biodiversity. In contrast, farmers are interested in maximising other ES and are induced to stop mowing by other contextual factors including profitability or topography. The ES framework could help design public policies to reconcile the interests of different stakeholders.

## Conclusion

To our knowledge this is the first ES study exploring the feedback between multiple ES and stakeholders' behavior through the decisions making process. By demonstrating the causal chain and mechanisms leading farmers to adopt a behavior, our study shows that farmer's knowledge about ES and willingness to benefit from these services are taken into account in their decisions, but do not constitute the main factor of decision. ES constitute necessary but not sufficient conditions in explaining behaviors, as other key factors were determinant (i.e. altitude, topography, parcels location, policies or social value). Such an approach should be tested at other sites with a greater set of ES and/or other beneficiaries and land managers, as well as in other natural resource management systems.

## Supporting Information

Figure S1 (a) Study site map with grassland management types and location of farms and roads (modified from [62]). Maps made by farmers during the 2009 interviews: (b) farmers' expectations about forage quality and quantity (colours) for mown (plain) or grazed parcels (shaded); (c) current practices and (d) current date of mowing.
(TIF)
