# Supplementary figures and images for: How Ecosystem Services Knowledge and Values Influence Farmers' Decision-Making
Source: PLoS One. 2014 Sep 30;9(9):e107572. doi: 10.1371/journal.pone.0107572 (PMC4182349; doi:10.1371/journal.pone.0107572)

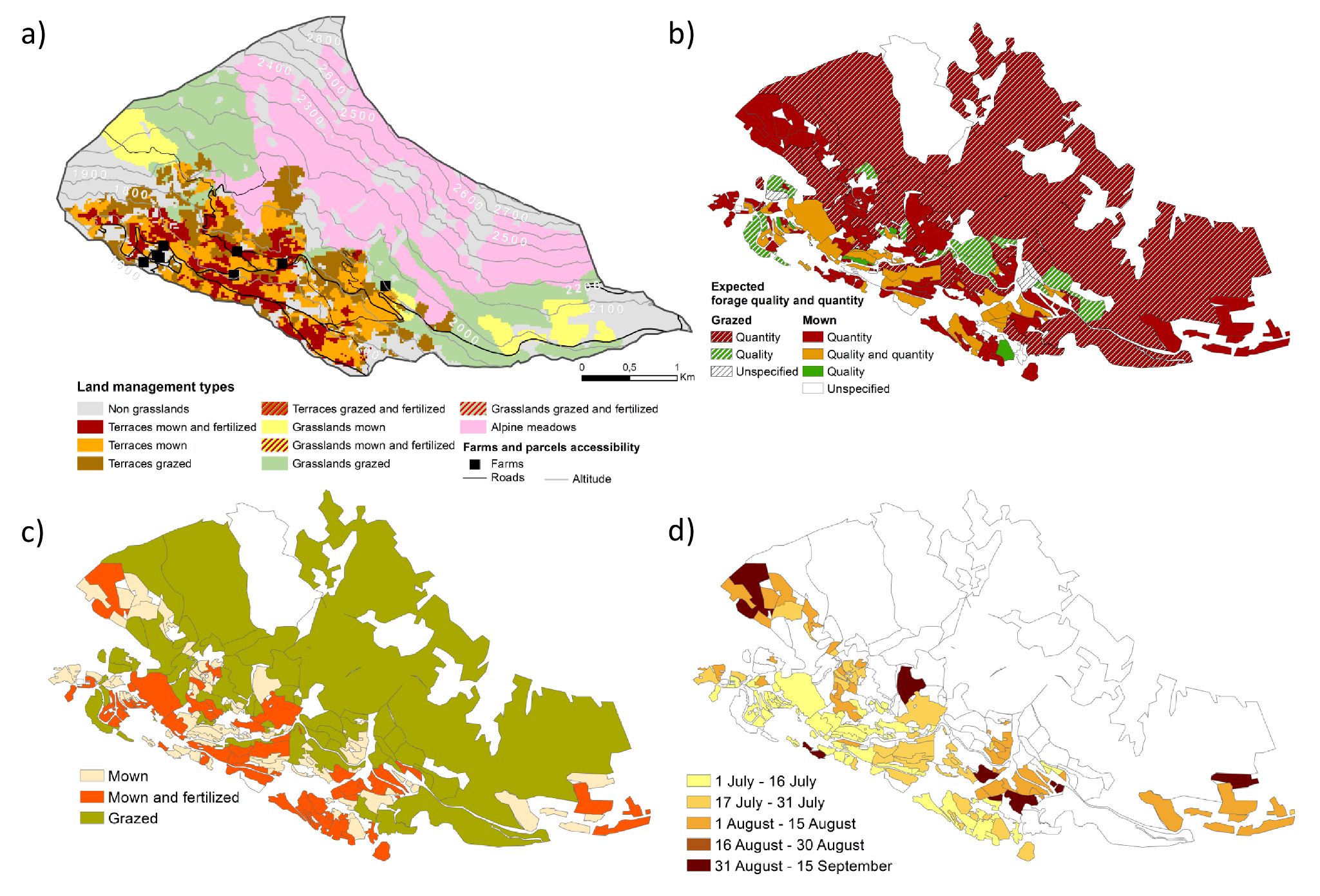

Supplement: Figure S1 — (a) Study site map with grassland management types and location of farms and roads (modified from [62]). Maps made by farmers during the 2009 interviews: (b) farmers' expectations about forage quality and quantity (colours) for mown (plain) or grazed parcels (shaded); (c) current practices and (d) current date of mowing. (TIF) [file pone.0107572.s001.tif]
